# Dosimetric Impact of Inter-Fraction Anatomical Changes in Carbon Ion Boost Treatment for High-Risk Prostate Cancer (AIRC IG 14300)

**DOI:** 10.3389/fonc.2021.740661

**Published:** 2021-09-28

**Authors:** Stefania Russo, Rosalinda Ricotti, Silvia Molinelli, Filippo Patti, Amelia Barcellini, Edoardo Mastella, Andrea Pella, Chiara Paganelli, Giulia Marvaso, Matteo Pepa, Stefania Comi, Mattia Zaffaroni, Barbara Avuzzi, Tommaso Giandini, Emanuele Pignoli, Riccardo Valdagni, Guido Baroni, Federica Cattani, Mario Ciocca, Barbara Alicja Jereczek-Fossa, Ester Orlandi, Roberto Orecchia, Barbara Vischioni

**Affiliations:** ^1^ Medical Physics Unit, Clinical Department, National Center for Oncological Hadrontherapy (CNAO), Pavia, Italy; ^2^ Bioengineering Unit, Clinical Department, National Center for Oncological Hadrontherapy (CNAO), Pavia, Italy; ^3^ Radiotherapy Unit, Clinical Department, National Center for Oncological Hadrontherapy (CNAO), Pavia, Italy; ^4^ Division of Radiotherapy, IEO, European Institute of Oncology Istituto di Ricovero e Cura a Carattere Scientifico (IRCCS), Milan, Italy; ^5^ Department of Electronics, Information and Bioengineering, Politecnico di Milano, Milan, Italy; ^6^ Department of Oncology and Hemato-oncology, University of Milan, Milan, Italy; ^7^ Medical Physics Unit, IEO, European Institute of Oncology Istituto di Ricovero e Cura a Carattere Scientifico (IRCCS), Milan, Italy; ^8^ Department of Radiation Oncology, Fondazione Istituto di Ricovero e Cura a Carattere Scientifico (IRCCS) Istituto Nazionale dei Tumori, Milan, Italy; ^9^ Medical Physics Unit, Fondazione Istituto di Ricovero e Cura a Carattere Scientifico (IRCCS) Istituto Nazionale dei Tumori, Milan, Italy; ^10^ Scientific Directorate, IEO, European Institute of Oncology Istituto di Ricovero e Cura a Carattere Scientifico (IRCCS), Milan, Italy

**Keywords:** carbon ion radiotherapy (CIRT), high-risk prostate cancer, image-guided radiotherapy (IGRT), inter-fraction anatomical changes, dose-of-the-day calculation, deformable image registration (DIR)

## Abstract

Rectum and bladder volumes play an important role in the dose distribution reproducibility in prostate cancer adenocarcinoma (PCa) radiotherapy, especially for particle therapy, where density variation can strongly affect the dose distribution. We investigated the reliability and reproducibility of our image-guided radiotherapy (IGRT) and treatment planning protocol for carbon ion radiotherapy (CIRT) within the phase II mixed beam study (AIRC IG 14300) for the treatment of high-risk PCa. In order to calculate the daily dose distribution, a set of synthetic computed tomography (sCT) images was generated from the cone beam computed tomography (CBCT) images acquired in each treatment session. Planning target volume (PTV) together with rectum and bladder volume variation was evaluated with sCT dose-volume histogram (DVH) metric deviations from the planning values. The correlations between the bladder and rectum volumes, and the corresponding DVH metrics, were also assessed. No significant difference in the bladder, rectum, and PTV median volumes between the planning computed tomography (pCT) and the sCT was found. In addition, no significant difference was assessed when comparing the average DVHs and median DVH metrics between pCT and sCT. Dose deviations determined by bladder and rectum filling variations demonstrated that dose distributions were reproducible in terms of both target coverage and organs at risk (OARs) sparing.

## Introduction

Essential issues in prostate cancer adenocarcinoma (PCa) irradiation are prostate motion and shape variations due to rectum and/or bladder filling modifications (1), which may strongly affect the target dose distribution and Organs at Risk (OAR) sparing (2). In order to maintain consistent rectum and bladder volume throughout the treatment, preparation instructions about food and fluid intake are usually given to each patient before treatment simulation and delivery. Despite this, inter-fractional unpredictable OAR volume variation might occur, and the reproducibility of dose distribution remains essential to providing an adequate and safe treatment of patients.

In this context, image-guided radiation therapy (IGRT) is essential to ensuring treatment efficacy and safety. In recent years, the introduction of new advanced techniques of IGRT using online cone beam computed tomography (CBCT) allows the tracking of daily positioning and anatomical changes of patients in treatment position. It also has the potential to be used to evaluate the dose-of-the-day distributions in comparison to the dose distribution calculated on the planning computed tomography (pCT) (3).

Since 2016 at the Centro Nazionale di Adroterapia Oncologica (CNAO, Pavia, Italy), we have enrolled patients in the phase II clinical trial with a mixed-beam approach for prostate irradiation, in collaboration with Istituto Europeo di Oncologia IRCCS (IEO) and Fondazione IRCCS Istituto Nazionale dei Tumori (INT) in Milan, Italy. The irradiation scheme consisted of a hypo-fractionated carbon ion radiotherapy (CIRT) anticipated boost to the prostate, followed by photon intensity modulated radiation therapy (IMRT) to the prostate and pelvic lymph nodes (grant AIRC IG 14300) (4).

The rationale of the hypo-fractionated CIRT boost is to escalate the biological dose to the target by exploiting carbon ion favorable physical and biological properties. The higher radiobiological effectiveness (RBE) of carbon ions on cancer radioresistant clones and more hypoxic tumor components (5) should enhance the efficacy of the subsequent photon phase of the scheme, delivered with conventional fractionation. Safety and effectiveness data on CIRT are derived from Japanese experience, where CIRT has been employed for unresected PCa since 1995 at the National Institute of Radiological Sciences (NIRS, Chiba, Japan), with excellent clinical toxicity and efficacy outcomes (6, 7).

Our study aimed to evaluate the impact of bladder and rectum filling variations in the CIRT dose-of-the-day distribution of target and OARs in the context of our phase II mixed beam study for high-risk PCa. Additionally, our IGRT and patient preparation protocol reliability was assessed, evaluating the dose distribution reproducibility during the treatment course using CBCT data.

Dose evaluation on daily CBCTs for particle therapy is challenging due to increased scatter, beam hardening, Hounsfield unit (HU) inaccuracy and often small field-of-view (FOV) sizes (3). In this study, we proposed a method for dose-of-the-day calculation. Synthetic computed tomography (sCT) images were obtained by deforming pCT images into the daily CBCT frame of reference. Subsequently, the pCT Hounsfield units (HUs) were transferred to sCT to obtain the corresponding stopping power maps for CIRT dose calculation.

## Material and Methods

### Patient Cohort

We retrospectively analyzed treatment and imaging data of 16 consecutive patients, enrolled from 2016 to 2020 in a phase II study for CIRT boost treatment at CNAO, diagnosed with high-risk PCa according to the inclusion criteria previously described in Marvaso et al. (4). Patients’ enrollment started after trial approval from all treating centers’ Ethical Committees (8). The selected patients underwent carbon-ion boost at CNAO, followed by photon intensity-modulated radiotherapy (IMRT) at IEO or INT, and signed informed consents at the coordinating center prior to treatment. Daily imaging data sets of two patients were incomplete and excluded from the study.

### Target Definition and Treatment Planning

The simulation CT acquired at CNAO was registered with the magnetic resonance (MR) image set for clinical target volume (CTV) delineation. The CTV included the prostate and the proximal third of the seminal vesicles. According to the protocol, planning target volume (PTV) was created by adding safety margins to the CTV, 5 mm in all directions. Rectum, bladder, bowel, and femoral heads were contoured as OARs for plan optimization with the following constraints for the boost phase: rectum D_0.03cm3_ ≤ 100%, V_16Gy(RBE)_ ≤ 5%, V_15Gy(RBE)_ ≤ 20%, bladder D_0.03cm3_ ≤ 102%, V_15Gy(RBE)_ ≤ 35%, femoral head V_10Gy(RBE)_ ≤ 15%, and bowel V_16.6Gy(RBE)_ = 0%. Target coverage objectives were PTV D_98%_ ≥ 95%, D_0.03cm3_ ≤ 107%, and median dose ≤ 102%. Priority was given to OAR dose constraints over PTV coverage for boost plans. More details on the cumulative plan acceptance criteria can be found in Gugliandolo et al. (8).

A total dose of 16.6 Gy (RBE) in four fractions (4.15 Gy (RBE)/fraction, over 1 week) was delivered for the CIRT anticipated boost at CNAO. Afterward, patients received a whole-pelvis IMRT of 50 Gy in 25 fractions at IEO or INT. In this study, only the CIRT treatment phase was considered.

Since no gantry was available, two opposed lateral beams were delivered using a fixed horizontal line and rotating the couch. This beam orientation was chosen to avoid placing the rectum and bladder distally from the beam (9), where range uncertainties can strongly degrade the dose distribution (10). The planned dose was delivered with pencil beam scanning technique with lateral spot spacing and energy layer spacing of 2 mm.

Treatment plans were optimized with RayStation v8.1 Treatment Planning System (TPS, RaySearch Laboratories Stockholm, Sweden). In order to mitigate range and setup uncertainties, a robust planning strategy was used based on minimax optimization, with setup and range uncertainties of 2 mm in all directions and 3%, respectively (11). The RBE-weighted dose was determined according to the local effect model LEM I (12) with an ideal α/β ratio of 2 Gy.

### Patient Positioning

For each treated patient, a pCT was acquired on a SOMATOM Sensation Open CT scanner (Siemens Medical Systems, Germany) using a slice thickness of 2 mm with a pixel spacing of 0.98 × 0.98 mm with machine parameters varying in the interval of 190–300 mAs at 120–140 kV.

During the pCT acquisition and the whole treatment course, all patients were immobilized in supine position with a pelvic personalized solid thermoplastic mask (Renfu Medical Equipment, Guangzhou, China) fixed on an indexed base plate. In addition, customized large cushions (TOTIM^®^ Patient Cushions Immobilization System, Essebi Medical SRL, Faetano, San Marino) were used in combination with knee and foot holders. An MR scan (Siemens Medical Systems, Germany) in the same setup condition was acquired after the pCT.

Patients were asked to empty the rectum with two micro enemas and drink 500 ml of water after bladder voiding, 30 minutes before starting the CT examination and before each treatment fraction, in order to maintain consistency in rectum and bladder filling and have a comfortable position with the rigid mask.

Before treatment delivery, patient setup optimization was image-guided by acquiring double planar orthogonal kV images in the anteroposterior and right–left directions. The acquired images were aligned automatically (after a preliminary manual alignment when necessary) to the corresponding digitally reconstructed radiographs (DRRs). Subsequently, the six degrees of freedom robotic couch (13) compensated for the estimated translations and rotations. After patient setup adjustment (14) and before treatment delivery, a daily CBCT was acquired for soft tissue anatomical change inspection purposes. Each CBCT image was evaluated by a radiation oncologist (RO), and if rectum or bladder filling was considered inadequate for treatment, the patient was asked to repeat the preparation.

CBCT images were acquired with a non-isocentric, custom-designed robotic imaging system (15). About 600 projective images were acquired during 220° gantry rotation around the patient. CBCT acquisition parameters were set to 120 kVp and 31 mAs. According to this clinical workflow, the bony anatomy imaged in the CBCTs is intrinsically co-registered to the pCT. The validity of this assumption was assessed by computing the 3D–3D registration between CBCTs and pCT, which resulted in sub-millimeter/degree of setup residuals.

CBCT volumetric images were reconstructed with a spatial resolution of 1 × 1 × 1 mm and stored in Meta Image file format (text-based tagged file format ".mha"). For this study, the axial field of view (FOV) of the CBCT was reduced to a diameter of 200 mm in order to mitigate the truncation artifacts. In addition, CBCTs were converted in DICOM format with the same frame of reference of the planning CT, for loading into the RayStation TPS.

### Synthetic CT of the Day and Dose Recalculation

**Figure 1:**
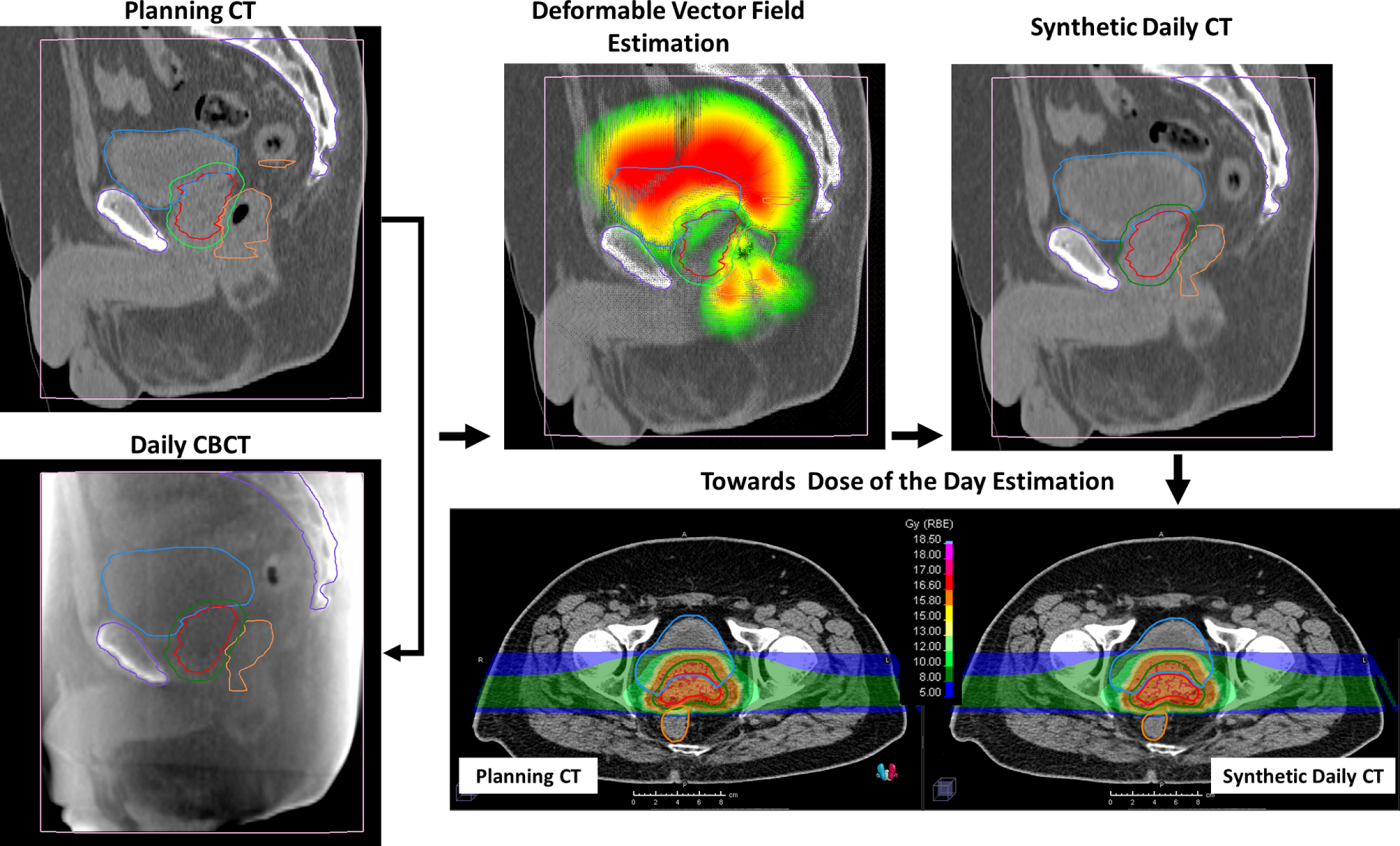
Representative example of the study workflow. Bladder (blue), rectum (orange), clinical target volume (CTV) (red), and planning target volume (PTV) (green) were contoured both on planning computed tomography (pCT) and on cone beam computed tomography (CBCT). Bony anatomy (violet) was delineated in pCT and rigidly propagated on CBCTs. The deformation vector field was estimated considering the delineated structures as “controlling ROIs,” focusing on the CBCT field of view. The planned dose distribution was then recalculated on the resulting synthetic CT (sCT).

The sCTs were created by deforming the initial pCT (*target image set*) on each daily CBCT (*reference image set*) by using the ANAtomically CONstrained Deformation Algorithm (ANACONDA) implemented in RayStation TPS (16). We mainly exploited the structure-based approach of the algorithm discarding the image intensity information during algorithm computation and focusing the deformation on the CBCT FOV. At first, femur heads and other pelvic bones, including sacrum and coccyx, contoured as landmarks on the pCT, were cropped according to the CBCT FOV. Subsequently, these contours were rigidly transferred on each CBCT. CTV, PTV, rectum, bladder, and bowel were manually re-contoured on each CBCT for each patient by an in-training RO and verified by an experienced RO. PTV, rectum, bladder, and the FOV-cropped bony structures were used as controlling regions of interest (ROIs) to drive the deformation. The deformation vector field was estimated with a resolution comparable to pCT of 1 × 2 × 1 mm/voxel in right–left, inferior–superior, and posterior–anterior directions, respectively. Each resulting deformation vector field was exported from RayStation and applied to the pCT using Plastimatch (version 1.9.0), yielding a sCT. The resulting sCT mimicked the original pCT outside the CBCT FOV, where the deformation vector field was zero, while inside the FOV the pCT was deformed according to the soft tissue geometry as detected in the CBCT. The planned dose distribution was recalculated on each sCT to have a reasonable estimation of the patient delivered dose on the anatomy of each treatment session. The resulting sCTs were evaluated in terms of quality of the deformation, analyzing the correspondence of bladder, rectum, prostate, and bones position between sCTs and CBCTs and confirming the absence of the deformation field outside the CBCT FOV. A schematic representation of the method described here to generate a sCT for dose-of-the-day recalculation is depicted in **Figure 1**.

### Data Analysis

Inter-fractional changes in patient anatomy were estimated computing bladder, rectum, and PTV volume variations between planning and treatment fractions. The coefficient of variation (CV) of the structure volumes was investigated for the whole patient cohort and each patient separately.

Average DVHs were obtained for the PTV, rectum, and bladder for pCT and sCT dose distributions, and the standard deviation of the population was computed at each dose level.

Treatment plan dose constraints for the bladder and rectum, together with PTV coverage objectives, were verified on each recalculation plan. The following DVH-based metrics were extracted for the pCT and each sCT: V_5Gy(RBE)_, V_10Gy(RBE)_, V_15Gy(RBE)_, and V_16Gy(RBE)_ for rectum and bladder, and D_95%_, D_98%_ D_50%_, D_2%_, and D_0.03cm3_ for PTV. sCT metric deviations from the planning values were evaluated with the Wilcoxon signed-rank test.

Finally, correlations between the bladder and rectum volumes, and the corresponding DVH metrics, were evaluated with the Spearman correlation test.

## Results

A total of 56 CBCTs were evaluated: 4 daily CBCTs for each of the 14 enrolled patients.

**Figure 2:**
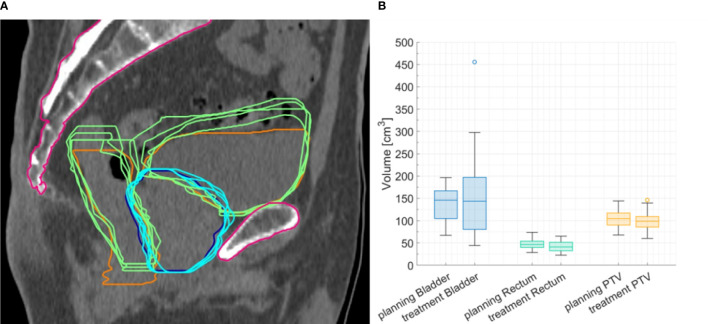
**(A)** Representative planning image with planning computed tomography (CT) contours of planning target volume (PTV) (blue line) and rectum and bladder (orange lines), with contours delineated on cone beam CTs (CBCTs) superimposed (light-blue and green lines). **(B)** Boxplots for bladder, rectum, and PTV volume at planning CT and CBCT for the whole cohort.

Volumes of bladder and rectum varied across patients and fractions. **Figure 2A** shows the bladder, rectum, and PTV simulation contours on the planning CT, with the contours derived from the daily CBCTs superimposed, for P7, as an example. For bladder, a higher-volume variability across patients and treatment fractions was found (ranging from 44 to 455.5 cm^3^, CV: 54.2%), as compared to rectum (ranging from 22.9 to 65.3 cm^3^, CV: 25.4%) and PTV (ranging from 60.2 to 146.5 cm^3^, CV: 21.0%). **Figure 2B** shows the distribution of organ volumes at pCT and during the treatment course, considering the whole patient cohort. According to the Wilcoxon test, there was no significant difference in the bladder, rectum, and PTV median volumes between the pCT and the CBCTs acquired during the treatment course (**Table 1**).

**Table 1:** Comparison of volumes and dose–volume histogram (DVH) indices of bladder, rectum, and planning target volume (PTV) at planning CT (pCT) and at synthetic CTs (sCTs) for all the patients.

		Bladder			Rectum		
		Planning	Treatment	Wilcoxon test	Planning	Treatment	Wilcoxon test
		median (IQR)	median (IQR)	p-value	median (IQR)	median (IQR)	p-value
**Volume**	**cm^(3)^ apex**	146.3 (62.3)	143.5 (116.6)	0.924	46.5 (14.6)	40.8 (18.8)	0.125
**DVH metrics**	**V16 Gy(RBE) [%]**	14.1 (6.5)	12.6 (10.0)	0.659	4 (1.1)	2.8 (2.8)	0.165
	**V15 Gy(RBE) [%]**	17.5 (9.0	16.1 (11.8)	0.654	11.9 (7.4)	9.8 (9.1)	0.336
	**V10 Gy(RBE) [%]**	26.8 (10.9)	27.1 (16.0	0.724	28.7 (11.5)	26.5 (14.2)	0.463
	**V5 Gy(RBE) [%]**	35.2 (13.8)	33.9 (20.8)	0.774	36.2 (11.4)	35.2 (19.1)	0.592
			**PTV**		
		**Planning**	**Treatment**	**Wilcoxon test**			
		**median (IQR)**	**median (IQR)**	**p-value**			
**Volume**	**cm^(3)^ apex**	104.8 (26.7)	98.5 (23.9)	0.45			
**DVH metrics**	**D98% [%]**	96.5 (1.9)	95.6 (3.0)	0.148			
	**D95% [%]**	98.6 (0.9)	98.2 (2.4)	0.354			
	**D50% [%]**	99.8 (0.3)	99.8 (0.3)	0.595			
	**D2% [%]**	100.6 (0.3)	100.6 (0.3)	0.699			

**Figure 3:**
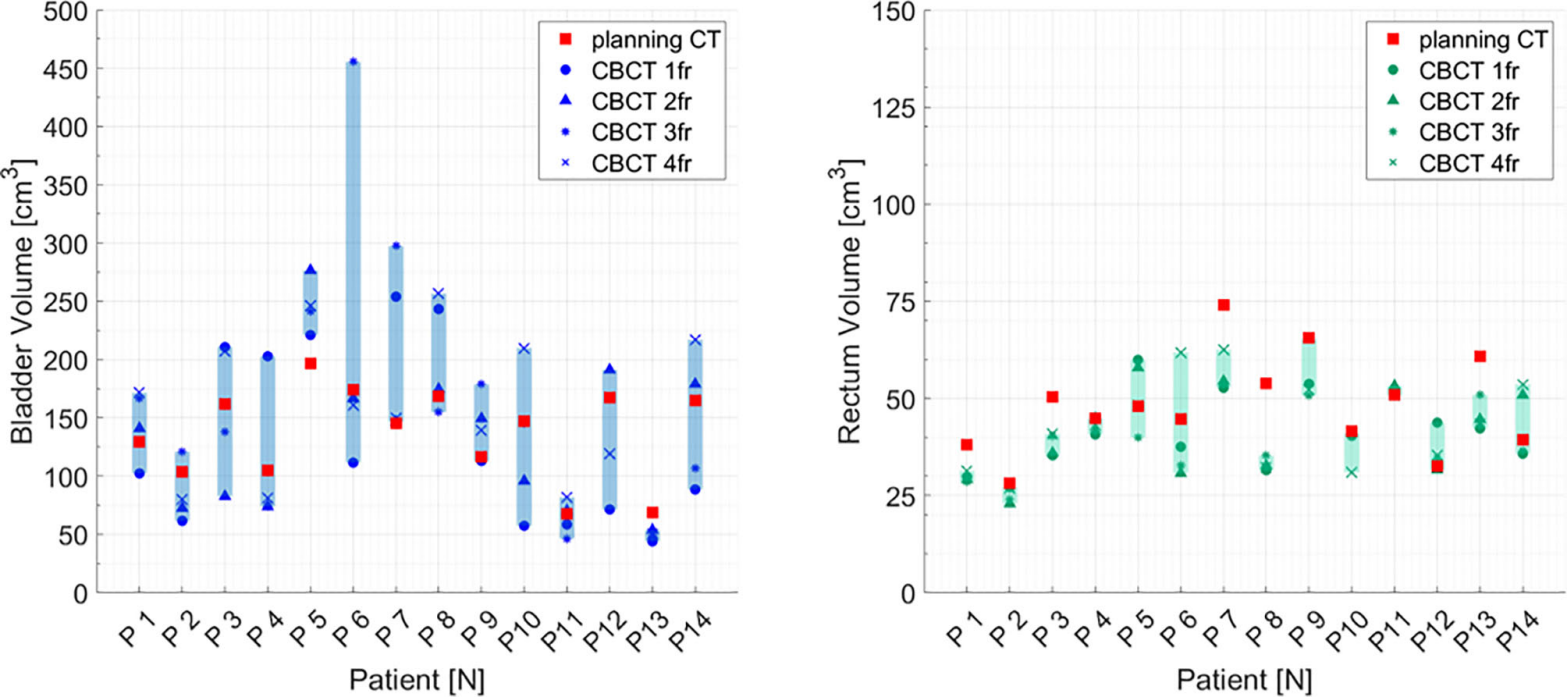
Bladder and rectum volume at planning CT (red squares) and at each cone beam CT (dark symbols) for all the patients separately. Graphical shades indicate the extent of volume variability.

Considering each patient separately, the volumes of bladder and rectum on the CBCTs were different compared to the volume on the pCT. Volume variations during the CIRT course were mostly patient-dependent: CV of bladder volume ranged from 8% for P13 to 70% for P6. Similarly, CV for rectum varied between 2% for patient P11 to 35% for P6 (**Figure 3**).

**Figure 4:**
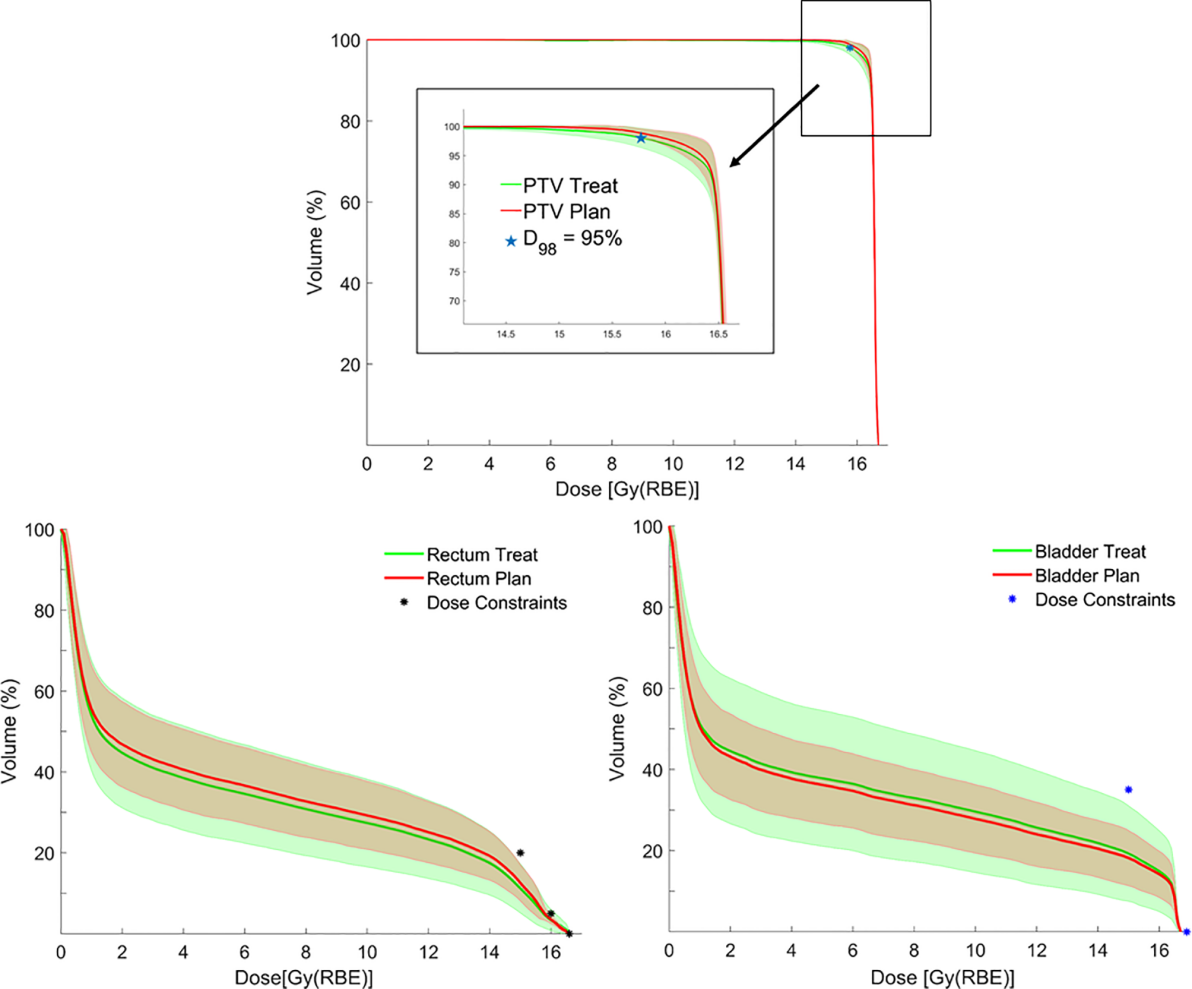
Average dose–volume histograms (DVHs) for planning target volume (PTV), rectum and bladder calculated on planning CTs (pCTs) (red lines), and synthetic CTs (sCTs) (green lines), with corresponding error bands (± 1 standard deviation) together with bladder/rectum dose constraints and PTV coverage objective.

In addition, no significant difference was found when comparing planned and treatment average DVHs as in **Figure 4**, and median DVH metrics between pCT and CBCTs for rectum, bladder, and PTV (**Table 1**).

**Figure 5:**
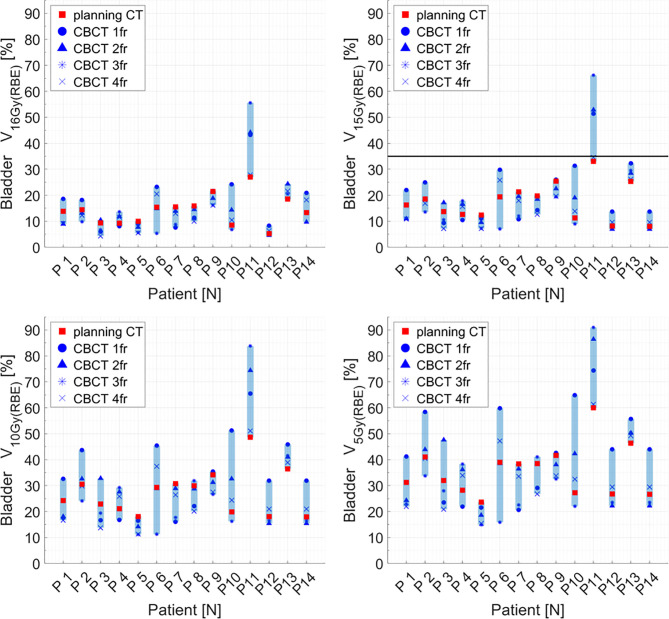
Distribution of bladder dose–volume histogram (DVH) metrics (V_16Gy(RBE)_, V_15Gy(RBE)_, V_10Gy(RBE)_, V_5Gy(RBE)_) for each patient at planning CT (red squares) and at each synthetic CT (sCT) (dark markers). Colored range bars indicate the maximum variation extent within a single patient. The bladder constraint V_15Gy(RBE)_ ≤35% is indicated as a black line. CBCT, cone beam computed tomography; fr, fraction.

**Figure 6:**
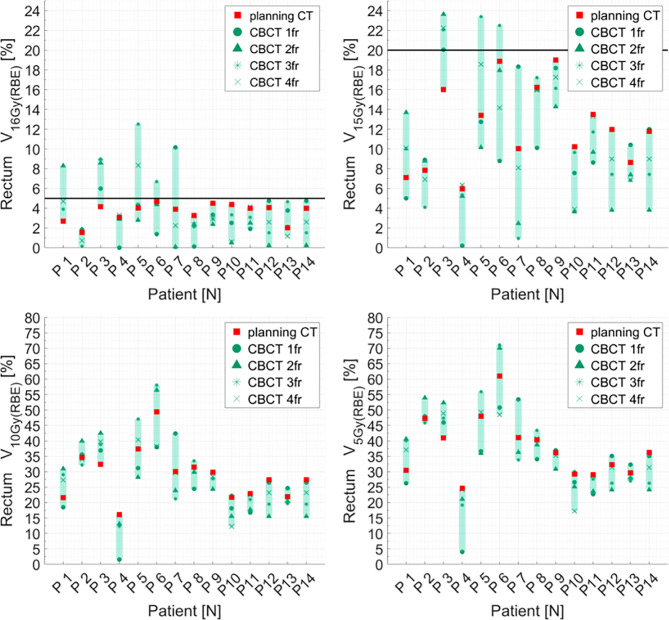
Distribution of rectum dose–volume histogram (DVH) metrics (V_16Gy(RBE)_, V_15Gy(RBE)_, V_10Gy(RBE)_, V_5GY(RBE)_) for each patient at planning CT (red squares) and at each synthetic CT (sCTs) (dark markers). Colored range bars indicate the maximum variation extent within a single patient. The rectum constraints V_16Gy(RBE)_ ≤5% V_15Gy(RBE)_ ≤20% are indicated as a black line. CBCT, cone beam computed tomography; fr, fraction.

**Figure 7:**
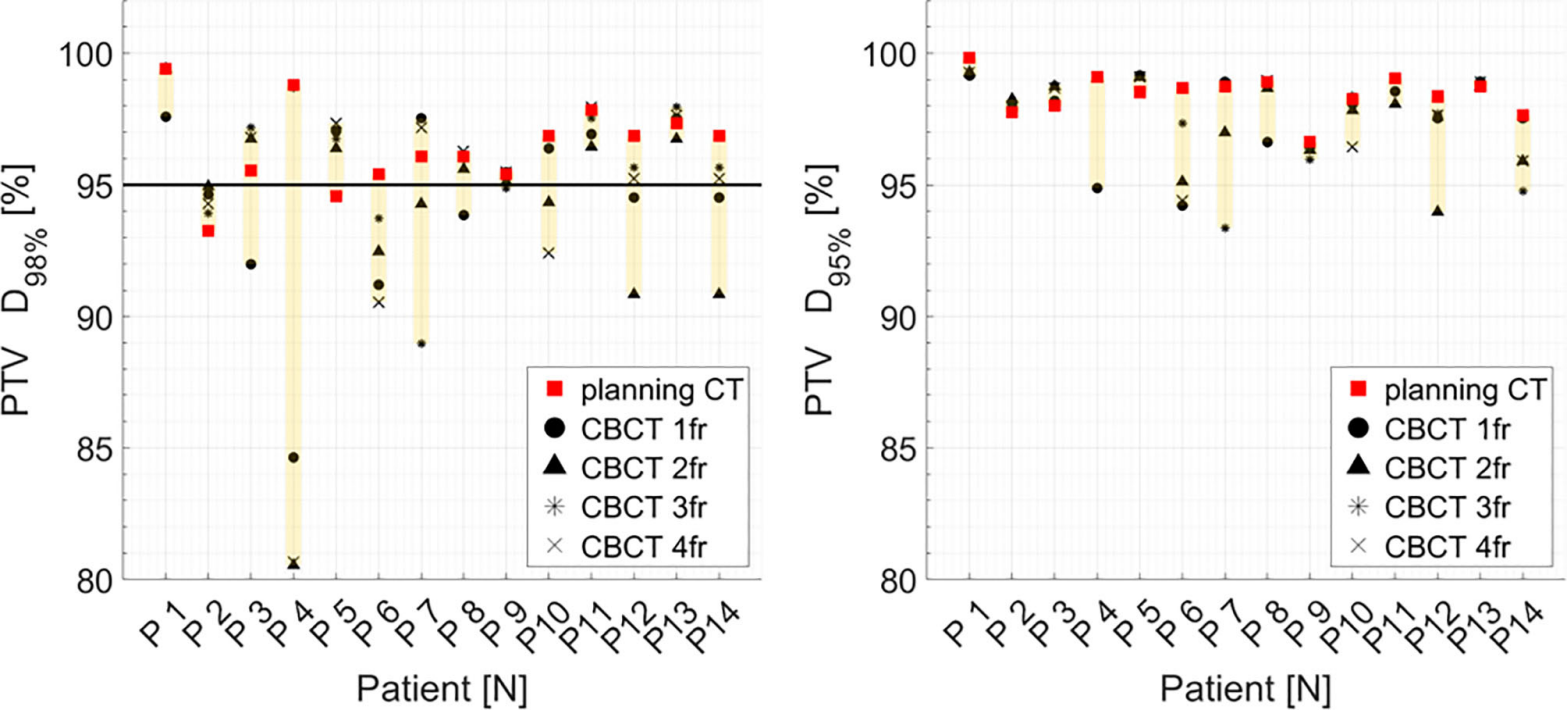
Distribution of PTV DVH metrics (D_98%_, D_95%_) for each patient at planning CT (red squares) and at each synthetic CT (sCTs) (dark markers). Colored range bars indicate the maximum variation extent within a single patient. Target coverage objective PTV D_98%_ ≥95% is indicated as a black line. CBCT, cone beam computed tomography; fr, fraction.

Distributions of DVH parameters for bladder, rectum, and PTV are presented separately for each patient in **Figures 5**–**7**, respectively. pCT treatment plans always satisfied all OAR constraints. Bladder V_15Gy(RBE)_ ≤ 35% was always met except for P11 for three out of four fractions. Rectum V_16Gy(RBE)_ ≤ 5% and V_15Gy(RBE)_ ≤ 20% dose constraints were met in 47 (84%) and 50 (80%) recalculation plans, respectively. In only one case (P3), rectum dose constraints were not met in all the recalculation plans. When considering high doses, no hot spots were found either in the rectum or in the bladder, with D_0.03cm3_ below 100% and 102% of the prescribed dose, respectively.

Median PTV D_98%_ and D_95%_ for treatment planning were not significantly higher compared to the sCT-recalculated plans (**Table 1**). The PTV coverage objective (D_98%_ ≥ 95%) was achieved in 12 of the 14 pCT optimized plans (85.7%), while this dose criterion was met in 34 of 56 (60.7%) cases in the recalculation plans (**Figure 7**). Concerning the hot spots, D_0.03cm3_ was lower than 107% of the prescription dose in all the cases.

In order to assess how bladder and rectum filling might affect OAR dose distribution, we considered these OARs’ overlap volumes with the 16-Gy (RBE) and 15-Gy (RBE) isodoses for each patient treatment plan. No correlation was found between the variation in rectum and bladder volume as compared to the pCT and the variation in the OAR overlap volume. In particular, rectum correlation coefficients were 0.19 (*p-value: 0.156*) and 0.34 (*p-value: 0.011*), respectively, whereas for bladder, correlation coefficients were 0.10 (*p-value: 0.444*) and 0.11 (*p-value: 0.425*).

On the contrary, the correlation between the absolute volumes of rectum and bladder with the respective absolute volumes receiving 16 Gy (RBE), 15 Gy (RBE), 10 Gy (RBE), and 5 Gy (RBE) increased as the considered dose decreases. For rectum, in particular, the correlation coefficient increased from 0.40 to 0.55 (*p-value <<0.001*), while for bladder the coefficient increased from 0.34 (*p-value: 0.0038*) to 0.61 (*p-value<<0.001*). The greater the OAR absolute volume, the greater the absolute volume receiving low doses.

A statistically significant correlation was found between the bladder absolute volume variation at pCT and CBCTs and the DVH metric variation at pCT and CBCTs expressed as a percentage of the corresponding volumes when considering the entire patient cohort. If bladder volume decreased during treatment, the percentage of bladder volume receiving 16 Gy (RBE), 15 Gy (RBE), 10 Gy (RBE), and 5 Gy (RBE) increased (correlation coefficient >0.5, *p-value <<0.001*).

## Discussion

The impact of inter-fractional variation in urinary bladder volume and rectum filling on daily dose distribution during CIRT for high-risk pCA was investigated in 14 patients enrolled in the AIRC IG 14300 grant frame (4).

For dose calculation on daily CBCT, we generate a sCT to overcome various CBCT limitations that forbid CIRT dose calculation. So far, sCT has been reported for proton dose calculations (17). This approach, based on Deformable Image Registration (DIR), has the advantage of not introducing HU inaccuracies in sCT images since the deformation does not modify the original pCT numbers. The distribution of HU between pCT and sCT was consistent for each contoured structure (bladder, rectum, PTV). Nonetheless, we are aware that this method has several issues that have to be addressed. Firstly, the air pockets eventually found in the CBCT images were propagated in the sCT, or conversely, when air pockets were found in the pCT they were not propagated in the sCT (18). However, considering the beam irradiation geometry, such areas were not included in the beam path. Secondly, the limited CBCT FOV resulted in incomplete patient external contour. Assuming that simulation CT was a reasonable estimation of the patient anatomy not included in the CBCT, pCT data were used to compensate for this missing information (19). Moreover, DIR could deform bones when large deformation occurs close to bony structures (20). To overcome any unrealistic bone deformations, we included bone ROI as a shape constraint during DIR computation.

One of the main goals of this study was to evaluate the validity of our IGRT approach consisting in bone-matching followed by CBCT acquisition. At present, the primary IGRT approach for moving targets, including pCA treatment positioning verification in particle therapy, consists in DRR bone-matching or target (prostate)-matching using implanted fiducials. Using orthogonal X-ray images for patient positioning verification is the standard procedure for CIRT in most centers (21). In our investigation, we found only very slight displacements of the PTV center of mass as determined by the contours on the sCT obtained from CBCT, with median (IQR) of 0.1(0.3)mm, -0.3(1.3)mm, and -0.3(0.5)mm toward the right, anterior, and inferior directions, respectively. These values were smaller than the prostate displacement found with IGRT techniques for photon IMRT prostate treatment (22). One possible explanation could be patient mask immobilization for CIRT, which might strongly reduce bowel and pelvic anatomy changes and thus prostate displacement (2). Looking at our results in terms of target displacement, our PTV margins (5 mm in all directions) appear suitable for the considered patient population.

Maeda et al. (23) reported that prostate-matching was more reliable than the bone-matching approach regarding rectum dose constraint adherence and target coverage in prostate proton therapy delivered with geometry of two opposed beams. At CNAO, thin golden filaments called Gold Anchor™ were investigated to assess the improvement in prostate position verification (24). Their visibility on both CT and radiographic images and the possible perturbation of the carbon ion beams were investigated through tests in an anthropomorphic phantom and turned out to be acceptable. In the future, we aim to implant the Gold Anchor™ seeds in some patients to perform further validation of our IGRT protocol, comparing prostate-matching versus bone-matching approach for patient positioning before CBCT acquisition.

Another essential purpose of the presented study was the validation of our patient preparation protocol. Bladder filling or rectal gas movement may influence the prostate position and therefore affect the target coverage. In parallel, the rectum and bladder could eventually receive unwanted hot spots if such OARs move in the high-dose treatment area resulting from the two-lateral opposed beam irradiation geometry.

At present, optimal rectum and bladder filling conditions for prostate external beam photon RT are still debated. In our study, patient preparation aimed at achieving a comfortable bladder filling compatible with pelvic mask compression and treatment duration while preventing major rectum and bladder volume variation compared to simulation conditions. Despite that precise fluid intake instructions were given to the patients, bladder volume varied considerably during the CIRT course in the analyzed patient cohort—as for P7, a patient with important obstructive urine retention. To a lesser extent, also the volume of the rectum varied among the fractions during the CIRT course. However, no statistically significant increase of the dose to OARs at treatment was observed.

Similarly, no major dose degradation in terms of PTV dose was observed. Even if the PTV D98% ≥ 95% goal failed in 40.7% of the recalculated plans, only slight deviations were found in D95% for the pCT optimized plans, with a mean relative difference of 0.1%, considering all the treatment fractions in the series of patients.

At CNAO, patients with high-risk localized prostate cancer are currently being treated either with a photon-CIRT mixed beam approach or with a full course of CIRT of 66.4 Gy (RBE) delivered in 16 fractions (4 days/week) to the prostate and seminal vesicles (25), according to Japanese experience (26, 27). Our findings showed that the setup and IGRT protocols described here appeared to be suitable also for patients treated with a full course of CIRT.

In our investigation, we focused exclusively on the residual inter-fraction anatomical variations after bony alignment and did not consider intra-fraction motion. However, several recent studies on cine-MR imaging extensively reported that prostate intra-fraction motion could affect the target dose distribution (28, 29). These studies concluded that 5-mm PTV margins were adequate to guarantee target coverage even during long-lasting treatments (>10 min). Since the presented CIRT prostate treatment time was approximately 2 min per beam and a rigid thermoplastic mask was used for patient immobilization, we do not expect the intra-fraction prostate motion to impact the dose distribution significantly.

We are aware that one of the limitations of the current analysis is the small patient cohort. However, the enrolment of patients in the phase II protocol described here is ongoing, and we aim to validate the current data in a larger patient series as soon as more patients are treated.

## Conclusion

The dosimetric impact of anatomical changes on CIRT was assessed in the context of our phase II mixed beam study for high-risk pCa patients. Dose deviations as determined by bladder and rectum filling variations demonstrated that the preparation protocol and the IGRT approach described here could generate reproducible dose distributions in terms of target coverage and OARs sparing.

The generation of sCTs from daily CBCTs for dose-of-the-day calculation in CIRT for high-risk pCA is clinically feasible. The proposed method is suitable for an adaptive treatment strategy providing a daily treatment plan based on the actual anatomy.

## Data Availability Statement

The raw data supporting the conclusions of this article will be made available by the authors, without undue reservation.

## Ethics Statement

The studies involving human participants were reviewed and approved by Comitato Etico Pavia, Fondazione IRCCS Policlinico San Matteo, Pavia, Italy, notification no. 20140025096. The patients/participants provided their written informed consent to participate in this study.

## Author Contributions

Conceptualization, SR, RR, and BV. Methodology, SR, RR BV, SM, and AP. Software, SR and RR. Validation, SR, RR, BV, SM, AP, FP, and MC. Formal analysis, SR, RR, SM, BV, AP, GB, and MC. Data curation, SR, RR, BV, SM, and AP. Investigation, SR, RR, SM, FP, AB, EM, AP, CP, GM, MP, MZ, SC, BA, TG, EP, RV, GB, FC, MC, BAJF, EO, and RO. Writing—original draft preparation, SR, RR, and BV. Writing—review and editing, SR, RR, SM, FP, AB, EM, AP, CP, GM, MP, MZ, SC, BA, TG, EP, RV, GB, FC, MC, BJ, EO, and RO. All authors contributed to the article and approved the submitted version.

## Funding

This study was partially supported by a research grant from Associazione Italiana per la Ricerca sul Cancro (AIRC IG 14300): “Carbon ion boost followed by pelvic photon radiotherapy for high risk prostate cancer”, registered at ClinicalTrials.gov (NCT02672449).

## Conflict of Interest

The authors declare that the research was conducted in the absence of any commercial or financial relationships that could be construed as a potential conflict of interest.

## Publisher’s Note

All claims expressed in this article are solely those of the authors and do not necessarily represent those of their affiliated organizations, or those of the publisher, the editors and the reviewers. Any product that may be evaluated in this article, or claim that may be made by its manufacturer, is not guaranteed or endorsed by the publisher.

## Abbreviations

PCa, prostate cancer adenocarcinoma; OARs, organs at risk; CNAO, Centro Nazionale di Adroterapia Oncologica; IEO, Istituto Europeo di Oncologia; IRCCS, INT Fondazione IRCCS Istituto Nazionale dei Tumori; CIRT, carbon ion radiotherapy; IMRT, intensity modulated radiation therapy; RBE, radiobiological effectiveness; IGRT, image-guided radiation therapy; CT computed tomography; CBCT, cone beam computed tomography; FOV, field of view; sCT, synthetic computed tomography; pCT, planning computed tomography; CTV, clinical target volume; PTV planning target volume; MR, magnetic resonance; LEM, local effect model; DRR, digitally reconstructed radiograph; RO, radiation oncologist; ROI, region of interest; DVH, dose volume histogram; CV, coefficient of variation.
